# Daily Diagnostic Quality Computed Tomography-on-Rails (CTOR) Image Guidance for Abdominal Stereotactic Body Radiation Therapy (SBRT)

**DOI:** 10.3390/cancers16223770

**Published:** 2024-11-08

**Authors:** Rachael M. Martin-Paulpeter, P. James Jensen, Luis A. Perles, Gabriel O. Sawakuchi, Prajnan Das, Eugene J. Koay, Albert C. Koong, Ethan B. Ludmir, Joshua S. Niedzielski, Sam Beddar

**Affiliations:** 1 Department of Radiation Physics, The University of Texas MD Anderson Cancer Center, Houston, TX 77030, USAlaperles@mdanderson.org (L.A.P.);; 2 Department of Gastrointestinal Radiation Oncology, The University of Texas MD Anderson Cancer Center, Houston, TX 77030, USA

**Keywords:** SBRT, liver, pancreas, CT, image guidance, motion management, CT-on-rails system

## Abstract

Background/Objectives: Stereotactic body radiation therapy (SBRT) for abdominal targets faces a variety of challenges, including motion caused by the respiration and digestion and a relatively poor level of contrast between the tumor and the surrounding tissues. Breath-hold treatments with computed tomography-on-rails (CTOR) image guidance is one way of addressing these challenges, allowing for both the tumor and normal tissues to be well-visualized. Using isodose lines (IDLs) from CT simulations as a guide, the anatomical information can be used to shift the alignment or trigger a replan, such that normal tissues receive acceptable doses of radiation. Methods: This study aims to describe the workflow involved when using CTOR for pancreas and liver SBRT and demonstrates its effectiveness through several case studies. Results: In these case studies, using the anatomical information gained through diagnostic-quality CT guidance to make slight adjustments to the alignment, resulted in reductions in the maximum dose to the stomach. Conclusions: High-quality imaging, such as CTOR, and the use of IDLs to estimate the doses to OARs, enable the safe delivery of SBRT, without the added complexity and resource commitment required by daily online adaptive planning.

## Simple Summary

Radiation therapy is becoming increasingly important in the treatment of liver and pancreatic tumors, particularly in situations where high doses of radiation can be delivered safely. However, there are several challenges to treating tumors in the abdomen, including poor visibility of the tumor and movements due to breathing and digestion. The typical imaging available at the time of treatment makes it difficult to see both the tumor and nearby portions of the digestive tract, which is particularly sensitive to radiation damage. This paper describes the workflow involved when using high-quality computed tomography imaging at the time of treatment, to ensure that the tumor is accurately targeted and normal tissues are avoided. This study shows that by using these images and the planned dose distribution, the dose to normal structures can be maintained below specified targets. With this technology and workflow, more patients can benefit from high-dose radiation treatment to the liver and pancreas.

## 1. Introduction

Liver and pancreatic cancers are both challenging to treat, with poor overall prognoses. The overall survival benefit of radiation for pancreatic cancer [1,2,3,4,5,6,7,8,9,10,11,12,13] has been the subject of debate, partially due to the prevalence of distant failures and the use of less advanced radiation techniques in some studies. However, local control benefits have been observed, particularly with sufficient dose escalation. Additionally, radiation allows some patients with borderline resectable disease to become candidates for surgery. The role of radiation in liver cancer [14,15,16,17,18,19] is similarly the subject of debate, with local control benefits being observed, especially with dose escalation. The local control offered by radiation therapy in regard to both cancers becomes more relevant as improvements are made to systemic treatments. With sufficient care in avoiding organs at risk (OARs), stereotactic and other hypofractionated dose regimes that allow for dose escalation have become important tools for treating these diseases.

There are a number of challenges facing liver and pancreatic stereotactic body radiation therapy (SBRT) that can limit the ability to safely escalate the radiation dose if they are not addressed properly [20]. The first is intra-fraction motion, due to respiration and motility of gastrointestinal (GI) organs [21,22,23,24,25]. A number of strategies exist to address respiratory motion, including the breath-hold (BH) technique, abdominal compression, free breathing gating, and four-dimensional computed tomography (4DCT)-based motion margins [22,24,26,27]. Another type of motion that should be addressed is inter-fraction motion, caused by changes in the stomach and bowel related to stomach filling and intestinal gas [28]. These changes could cause sensitive GI structures to move closer to the treatment field than they were during simulation, either through organ or tumor motion, potentially resulting in unanticipated damage if not properly accounted for.

**Figure 1:**
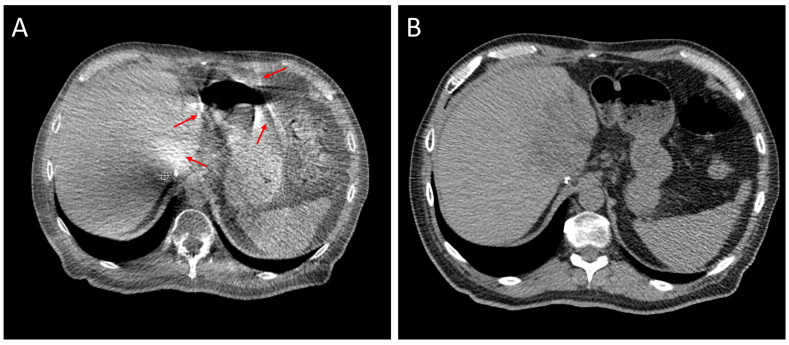
A comparison between (**A**) a BH CBCT image and (**B**) a BH CT-on-rails image of the same patient and anatomical location. Note the artifacts (red arrows) near the gas and high-density object in the CBCT image, which are not shown in the CTOR image. The boundaries of the stomach and bowel are also more clearly visualized in the CTOR image, compared to the CBCT image. Also visible in the CTOR image, are features within the liver itself.

Another challenge in regard to the abdomen is the limited soft-tissue contrast, especially between the tumor and the surrounding normal tissue. Iodinated contrast media can be used during simulation to define the gross tumor volume (GTV) [29], but daily contrast injections during treatment require the dilution of the contrast agent and adds time and complexity to the treatment process [30]. Gas within the abdomen can cause streak artifacts in cone-beam computed tomography (CBCT) images, further compounding the visibility challenges. These artifacts are more pronounced in free-breathing CBCT images, but are often still present in breath-hold images. Advanced imaging, such as diagnostic-quality CT or MRI, can allow for sufficient gross tumor volume (GTV) and normal tissue visualization to ensure proper alignment with the target and avoidance of OARs [31,32,33]. Without advanced imaging, the treatment margins may need to be increased or fiducials put in place to ensure target coverage [34,35] and the prescriptions may need to be adjusted to ensure that OARs do not exceed the dose tolerance thresholds [36]. A comparison is shown in Figure 1 between diagnostic-quality CT used for alignment and CBCT. This article will focus on the technical aspects of the use of a CT-on-rails (CTOR) system [37,38,39,40,41], utilizing BH gating to address the challenges to delivering a high dose of radiation to the abdomen, allowing a safe level of dose escalation that ensures the dose constraints in regard to radiosensitive GI organs are met. This study aims to describe the workflow involved and demonstrate the benefits and potential pitfalls related to the use of this technology through several case studies.

Daily online adaptive planning has been shown to allow for improved target coverage, while maintaining normal tissue constraints [36,42,43,44,45,46,47,48,49,50,51,52,53,54,55]. However, online adaptive systems can be time consuming and resource intensive, generally requiring extensive medical physics and physician involvement during treatment [56,57]. Such systems can also add complexity, as they often require a separate treatment planning system (TPS) and may face challenges relating to interfacing with outside records and systems verification. While online adaptive planning is generally ideal when dealing with the abdomen, it may not be feasible to implement in all treatment centers or for all patient populations. Offline adaptation can be used, but may cause treatment delays and add complexity and require more personnel resources. Additionally, further changes to the patient’s anatomy could again lead to OARs receiving higher doses of SBRT than expected. Offline adaptation is best employed when consistent changes in the anatomy are observed. Another strategy is to lower the prescription dose, such that the OARs will not receive a dose above the stipulated tolerance regardless of any anatomical changes. This decrease in treatment efficacy may be needed when OARs are not visible in the daily alignment images, due to image quality limitations. An intermediate solution is to use isodose line (IDL) contours, derived from the planning CT displayed on the daily image to identify and avoid any excessive doses to normal tissues. This strategy will be explored in this paper. A comparison of these strategies is presented in Table 1.

Overall, this paper aims to demonstrate the workflow involved when using CTOR-based daily imaging and planning CT-based isodose contours to improve target alignment and normal tissue avoidance, during abdominal SBRT and hypofractionated treatments. After a description of the workflow steps, three case studies are presented to demonstrate the alignment improvements that are possible when using the daily CTOR system.

## 2. Methods and Materials

The protocol described in this section was approved by the University of Texas MD Anderson Cancer Center institutional review board (PA14-0646). MD Anderson Cancer Center has two CTOR treatment rooms, with beam-matched linacs and matched CTs. The vast majority of liver and pancreatic SBRT and hypofractionated treatments at our institution are conducted using these machines, while standard fractionated treatments generally use CBCT alignment. The patients presenting here were simulated using breath-hold (BH) image gating and CTOR image guidance. For patients receiving abdominal SBRT at our institution, BH is preferred for patients that can tolerate it. With this motion management technique, the motion of both the OARs and the tumor can be controlled, allowing for a larger portion of the tumor to be safely treated with a high dose. Inhalation BH is typically used, as it is generally easier for patients to understand the instructions, but exhalation BH is considered when it is dosimetrically favorable. For patients that cannot tolerate holding their breath repeatedly, 4DCT is used and an internal GTV (IGTV) is created. A compression belt is considered for patients who cannot hold their breath, but have large tumor motion.

### 2.1. Simulation

Patients are scanned using a Philips Big Bore CT scanner (Philips, Andover, MA, USA). To improve consistency in terms of the stomach-filling and bowel position, patients are instructed prior to the simulation not to eat or drink anything (“nil per os”, abbreviated to NPO) for three hours before arriving and are given instructions on the appropriate diet and/or medications to manage gas. These preparations are maintained during the treatment to improve consistency. Patients are positioned in a long stereotactic cradle over a wingboard, with their hands above their heads, holding a T-bar. A Varian Medical Systems (Varian, Palo Alto, CA, USA) real-time position management (RPM) box is placed on the patient’s abdomen and they are instructed to hold a comfortable breath multiple times, before a gating window is set centered on their natural breath-hold position. After establishing the baseline breath window, visual feedback goggles are used to assist the patient in consistently reproducing this breath-hold level. After a free-breathing scan, at least one breath-hold scan without contrast is acquired, followed by 3 or 4 scans with the use of iodinated intravenous (IV) contrast media. The contrast scans are started 30 s after contrast media injection and are acquired back-to-back to capture different contrast phases. A planning image is chosen based on the breath-hold position and contrast phase with the best tumor visualization.

If prior non-contrast imaging suggests that there will not be an anatomical landmark to align with, then fiducials may be implanted near the tumor prior to simulation [58,59,60]. However, generally the tumor itself or a surrogate structure can be visualized on daily non-contrast CT images, so the added risk involving implantation into the patient is avoided. Potential surrogate structures include blood vessels, fissures within the liver, cysts, or the edge of the liver.

### 2.2. Treatment Planning

**Figure 2:**
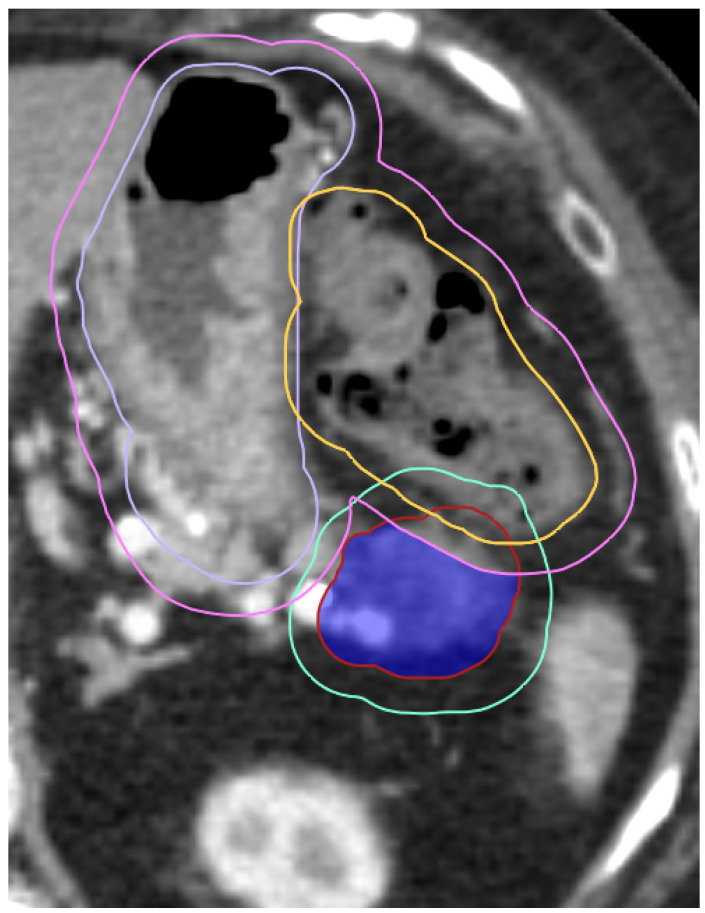
Example of an SIB approach to account for nearby radiosensitive OARs. In this case, the large bowel (orange) overlaps with the GTV (red) and the stomach (purple) is also close by. A GI PRV (pink) is created as an expansion of the GI luminal structures and is carved out from the high-dose PTV (shaded blue). An expanded low-dose PTV (teal) includes portions of the GTV that are not covered by the high-dose PTV.

To account for variations in the breath-hold position, an IGTV is contoured that includes the tumor position across all the breath-hold scans. A simultaneous integrated boost (SIB) approach is often utilized to allow for partial dose escalation, while protecting normal tissues [61,62]. The approach used at our institution is detailed in the work by Koay et al. [61] and is briefly described here. A PRV (planning OAR volume) expansion of 5 mm is added around all GI luminal structures and the heart. The high-dose planning target volume (PTV) is created as a margin around the IGTV subtracting the PRV and a lower dose PTV fills in the areas that have been carved out. Figure 2 demonstrates this technique in a patient, where their stomach and large bowel were close to the IGTV. Treatment planning is completed in RayStation, using the collapsed cone dose calculation algorithm. For OARs that have a maximum dose constraint, IDL contours are created and exported to be used during the alignment. The prescription dose levels are also exported as IDL contours.

### 2.3. Description of the CT-on-Rails System and Workflow

**Figure 3:**
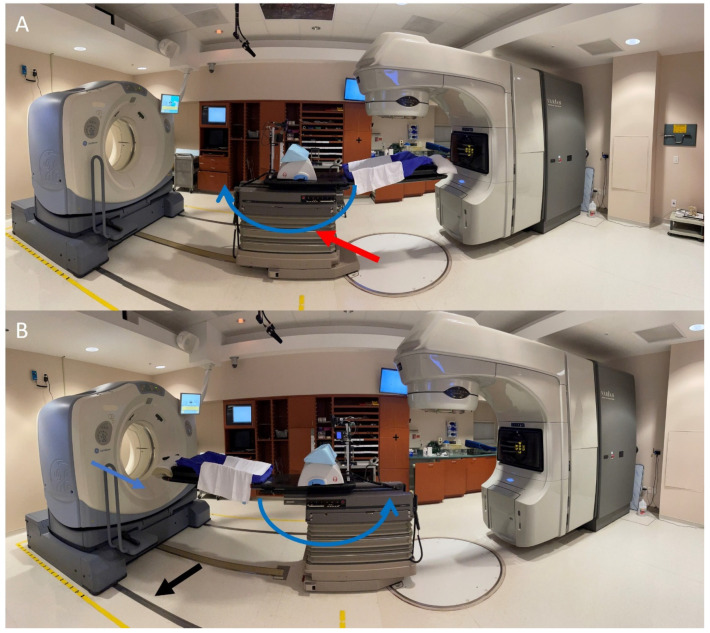
Photos of the CTOR–linac set up. First the patient is set up in the treatment position, with the couch positioned in the linac position (**A**) and BBs are placed in the laser positions. The base of the couch (red arrow) is then rotated 180° (curved blue arrows), according to the CT scanner position (**B**). The CT scanner moves along the rails on the floor (black arrow) in the direction of the straight blue arrow to take a scan. After image alignment, the patient is rotated back to the linac position and the lasers are aligned with the BBs. Final couch shifts are made from this position.

**Figure 4:**
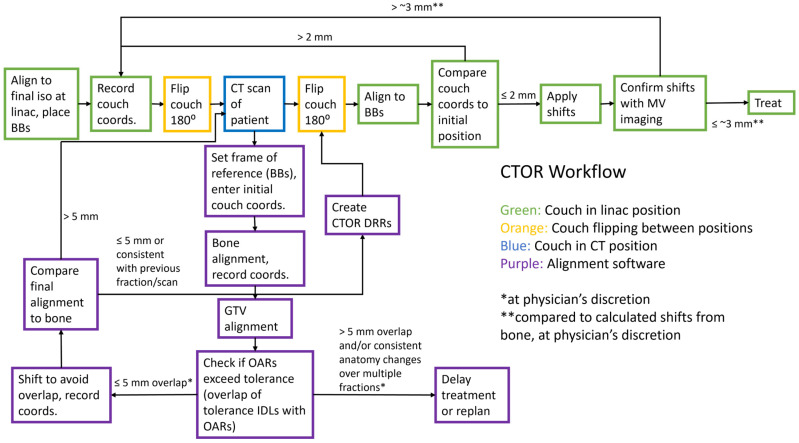
Flowchart of overall CTOR workflow (Section 2.3), including CTOR-based alignment and evaluation (Section 2.4) and verification imaging (Section 2.5).

The CT-on-rails delivery system consists of a GE CT scanner on rails, a Varian Clinac 21EX linac, and a couch that can rotate 180 degrees between the linac position and the CT scanning position (Figure 3). The overall workflow used for SBRT in patients treated with CTOR alignment is shown in Figure 4. The SBRT patients are treated during 40 min time slots, with an extra 10 min added on the first day. The patient is initially set up with the couch in the linac position. After the patient’s position within the cradle and rotation are verified in terms of free breathing, the patient is shifted to the final isocenter position. A gating window is set up, using the same window parameters as at the simulation (distance from normal breathing exhale to the bottom of the gating window and window width) and the patient is given feedback goggles to assist them in reaching the correct BH level. An RPM camera, attached to the end of the couch, is used so the camera is in the same position relative to the patient in both the linac and CT configurations. Radiopaque ball bearings (BBs) are placed on the patient when they are in the final isocenter position, while they are holding their breath, and are verified with a repeat BH. Since the CT and linac systems are separate systems, the BBs serve as the reference position in the CT scan to relate the coordinates back to the linac position. The initial couch coordinates are recorded and the patient is rotated into the CT position. A BH CT scan is then acquired with our GE LightSpeed 16 scanner (GE, Chicago, IL, USA), with a 0.98 × 0.98 mm^2^ pixel size, a 2.5 mm slice thickness, a 0.5 s rotation time, a 50 cm field-of-view, 120 kVp, and 250–350 mA (depending on the patient’s habitus and tumor contrast). Daily CT-on-rails images are aligned to the reference planning CT images using in-house software [63] (see next section), which calculates the final couch position from the inputted BB-aligned couch position. A rigid registration without rotation is performed, since the specialized rotating couch is not capable of 6 degrees-of-freedom motion. After image registration, the patient is rotated back to the treatment position and aligned to the BBs during a breath hold. The couch coordinates are compared to those initially recorded. A difference of 3 mm or more indicates that the patient has moved, which necessitates a repeat CT scan using the new BB position. Once the BB position is verified, the patient is shifted to the final couch position. Care is taken to verify the couch positions for multiple steps, since the manual entry of numbers is involved.

### 2.4. CT-on-Rails-Based Alignment

**Figure 5:**
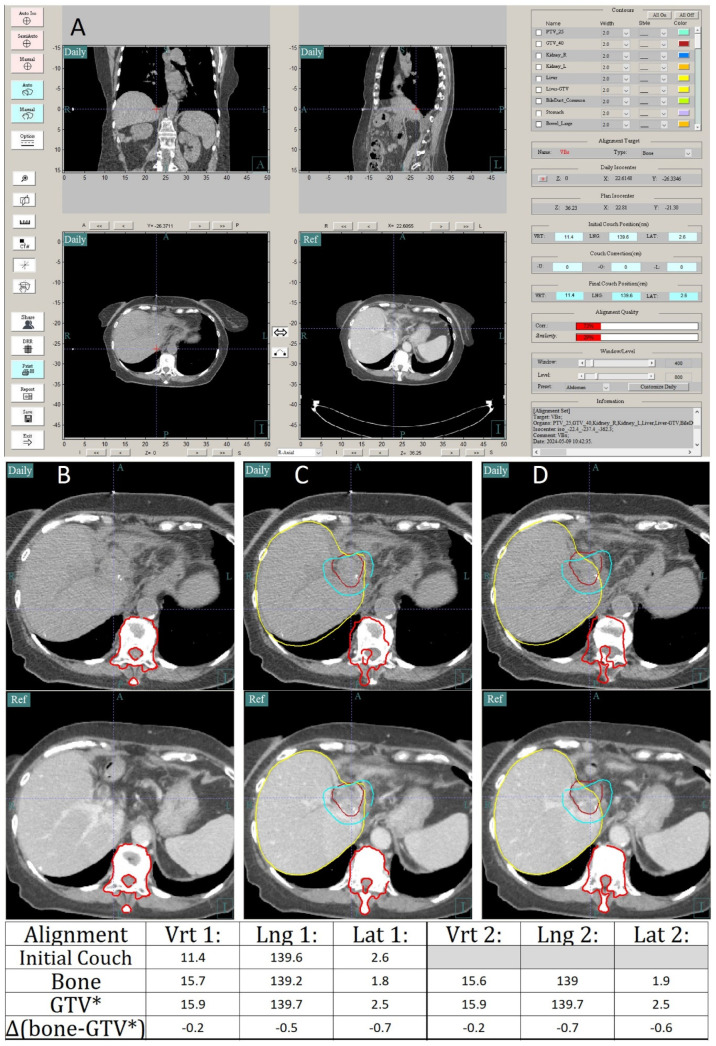
Example demonstrating the alignment workflow using in-house software for CTOR. A screenshot of the alignment software is shown in (**A**). As seen in (**A**), first the user aligns with the BBs to set the reference point and enters the initial couch positions, when the lasers were aligned to the BBs in the treatment position. Next, they align to the vertebral bodies (VBs) near the GTV, as shown in (**B**), with the daily CTOR image above and the CT simulation reference image below (red contour). Then, (**C**) they align to the GTV (dark red contour) and check that the desired IDL (teal contour) does not overlap with the relevant OARs. In this example, the difference between the bone alignment and GTV alignment is greater than 5 mm, as seen in alignment 1 in the chart at the bottom, so a second CTOR image is acquired. Again, the BB position is set and VB alignment is conducted, followed by GTV* alignment (**D**). The chart at the bottom of the figure shows the recorded couch positions (in cm) and differences between the bone and GTV* alignment for both scans. Since the differences between the bone and GTV* were within a few millimeters between the two scans, the couch positions from the second alignment were used as the treatment isocenter.

Daily CTOR images are aligned to the reference planning CT images, using the in-house software. First, a reference point is set at the location of the BBs in the CT scan. The longitudinal and vertical position are determined using one or both lateral BBs and the lateral position is determined using the anterior BB. Next, an alignment to the vertebral bodies near the GTV is performed, as a reference for how the internal anatomy has changed relative to bone. Then, the GTV is aligned to the planning CT. Any shifts away from GTV alignment to avoid OARs exceeding the tolerance dose (discussed in more detail below) are now made, resulting in what will be called GTV* alignment for clarity. A comparison is then made between bone and the GTV* alignment, in the spirit of creating action levels for large anatomical variations, as suggested by AAPM’s task group 101 on SBRT [64], and to help to detect outlier breath holds. If the difference between bone and the GTV* alignment is greater than 5 mm and the alignment is not consistent with previous fractions, subsequent CTOR images are acquired, until the GTV* position is consistent with the previous scan. If the patient’s positioning is demonstrated to be inconsistent by four CTOR images, the treatment will be aborted for the day and possibly replanned. An example of the CTOR alignment workflow is shown in Figure 5.

**Figure 6:**
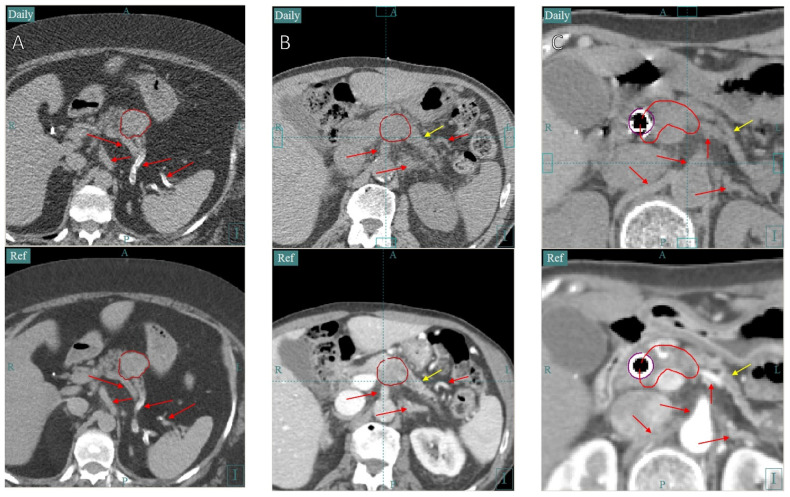
This figure demonstrates the ability to visualize with CTOR images the pancreas and the surrounding anatomy necessary to align with the GTV (red contour). The top row is the CTOR images and the bottom is the planning CT images. Comparing nearby blood vessels (red arrows) and the pancreas shape (yellow arrows) between the two images can be used to achieve good alignment in the superior–inferior direction, which is often the hardest, and can aide in the alignment in other directions as well. Calcifications within blood vessels (as seen in (**A**,**B**)) can also be a useful tool. Stents (purple contour in (**C**)) can be used to assist with alignment, but should be used with caution, as they may move relative to the tumor. The window/level for all the images is 400/800 HU.

**Figure 7:**
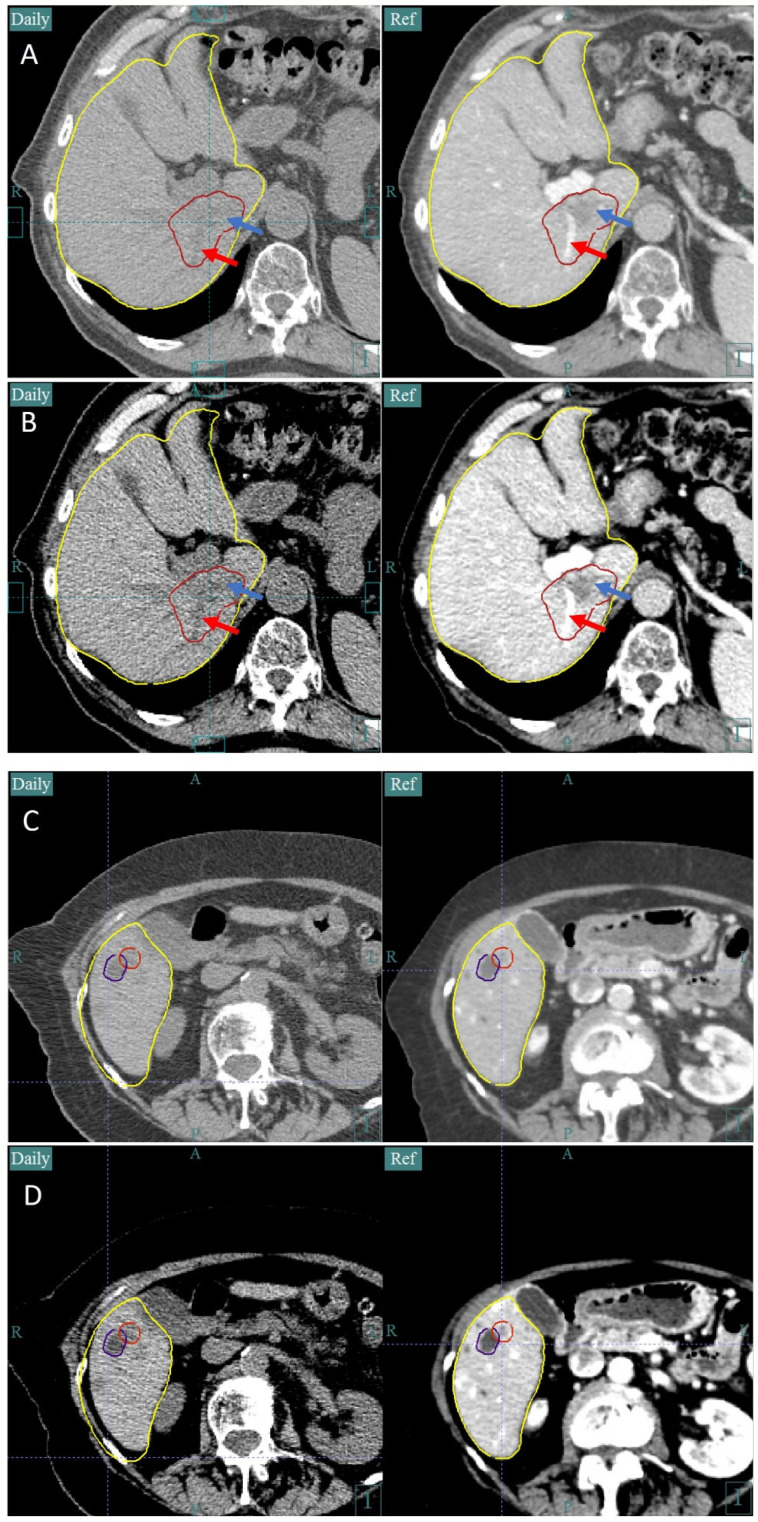
This figure demonstrates the ability to visualize the CTOR features within the liver necessary to accurately align with the GTV. In the left column are the CTOR images and in the right are the planning CT images. For the patient in (**A**), the liver shape (yellow contour), blood vessels, liver fissures in the vicinity of the tumor can be used to align to the GTV (red contour). Additionally, the tumor itself is somewhat visible (blue arrow), along with a blood vessel within the liver (red arrow). Using a different window and level (**B**) can improve the visualization of the tumor and interior blood vessel. In (**C**), the tumor itself is difficult to distinguish from normal liver, but a cyst (purple contour) right next to the tumor (red contour), along with the inferior liver shape, can be used to achieve accurate alignment. With a different window and level (**D**), the cyst becomes easier to distinguish and there is some distinction between the tumor and normal liver. In (**A**,**C**), a window level of 400/800 HU is used and, in (**B**,**D**), a window level of 180/950 HU is used.

Despite a lack of contrast agents for daily imaging, the GTV or a surrogate can generally be visualized in the CTOR image. Fiducial markers can be inserted in more difficult cases, but are not generally needed with CTOR. For tumors of the pancreas, surrogates such as blood vessels and the pancreas shape can be used when the tumor itself is difficult to visualize (Figure 6). For tumors of the liver, surrogates such as the nearby liver shape, blood vessels, fissures, or cysts can be used, when there is not enough contrast to visualize the tumor (Figure 7). Changing the CT image’s window and level can also help with visualizing the tumor itself (Figure 7). A window/level of 400/800 Hounsfield units (HU) is used for the visualization of organs at risk and 180/950 HU is used to visualize tumors and blood vessels in the liver. Prior to simulation, the physician should consider whether the tumor or a sufficient surrogate will be visible on non-contrast CT scans or whether fiducial markers should be inserted, using prior diagnostic non-contrast CT scans as a guide as to what should be visible when needed.

To assist with alignment evaluation, isodose lines are created in the TPS on the planning CT and transferred to the alignment software. Isodose lines for the maximum dose constraints of the structures near the GTV are displayed after GTV alignment; the daily image is then checked for overlaps between the maximum dose constraint isodose contours and the associated OARs. Typically for SBRT (5 fractions), the goal is for less than 1 cm^3^ of a given GI luminal structure to receive 35 Gy or higher, with a maximum of less than 40 Gy. However, these goals may vary depending on patient-specific factors, clinical trial enrollment, and anatomic consistency, among other factors. For slight overlaps (on the order of the PTV margin or smaller), the alignment can be shifted, such that the GTV contour is slightly misaligned in regard to the daily GTV position, but the IDL no longer overlaps the OAR. After slight GTV misalignment or for cases where the GTV is rotated or deformed relative to the simulation, sufficient GTV coverage can be evaluated using the prescription dose IDL. For larger overlaps, the treatment should be aborted and reattempted later in the day or the following day. For patients with repeated large overlaps, an adaptive plan could be created offline, using the daily CTOR image. Note that for the purpose of comparing bone to GTV alignment as described above, the final alignment (shifted slightly off GTV if needed for OAR sparing) is what is used (GTV*).

To demonstrate the appropriateness of using IDLs that were calculated on the planning CT and then transferred to the daily CT, the dose was recalculated in regard to the daily CTOR images for several patients and compared to the IDLs from the planning CT. Additionally, to demonstrate the appropriateness of shifting away from the GTV to satisfy maximum dose constraints related to the bowel, the dose was recalculated in regard to the daily CTOR images with and without this shift to compare GTV coverage and OAR doses. The results of these comparisons are detailed in Section 3.2 and Section 3.3.

### 2.5. Verification Imaging

Prior to each treatment, orthogonal digitally reconstructed radiographs (DRRs) from the CTOR image are calculated as a reference for the expected bony anatomy position relative to the daily treatment isocenter. To verify that the patient has not moved and that shifts have been applied correctly, orthogonal MV images are acquired. Both the MV images and CTOR-derived DRRs are compared to the DRRs from the planning CT. The MV images are expected to have the same shifts (within a few millimeters at the physician’s discretion) away from the bony anatomy alignment as is observed in the CTOR DRRs, which should numerically agree with the shifts between the bone and GTV alignment that were recorded during the alignment process. Any discrepancies warrant further investigation and likely a repeat of the CTOR scan in order to repeat the workflow.

## 3. Results

### 3.1. Case Study 1

**Figure 8:**
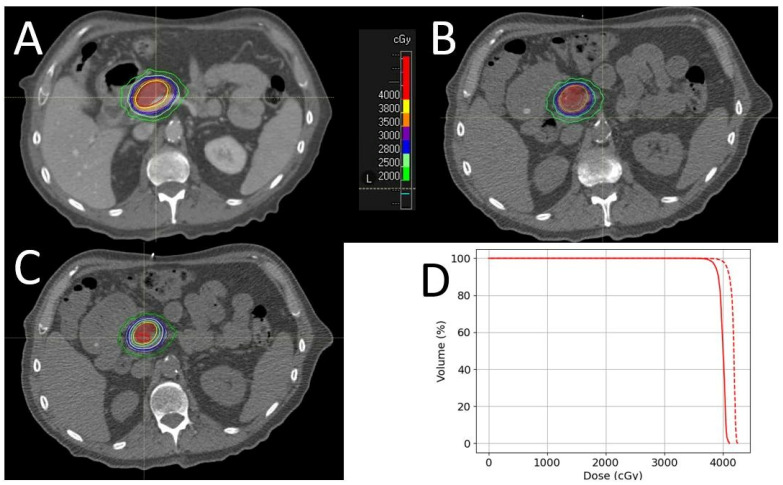
An example of the effect of gas bubbles on the planned vs. actual dose distribution. (**A**) Shows the treatment plan according to the simulation, with the GTV in red and various isodose levels shown; here, a large gas bubble is very close to the GTV. (**B**) Shows the day-of CTOR image with the original dose distribution, while (**C**) shows the day-of CTOR image with the dose recalculated to account for the now-absent gas bubble, showing a decrease in target coverage. (**D**) Shows the planned (dashed) vs. actual (solid) DVHs for the GTV for this fraction, extrapolated to five fractions, assuming the same dose is delivered to each fraction.

The first case study focuses on the effects of changes in bowel gas between the simulation and treatment. This patient with adenocarcinoma of the pancreas received SBRT to the pancreas, with a dose of 40 Gy in five fractions. They had a sizeable gas bubble close to the GTV in their stomach during the simulation, which resolved itself for part of their treatment. The fraction with the most dramatic difference in terms of the gas is illustrated in Figure 8. When the dose is recalculated in regard to the daily CTOR image without the gas, a decrease in the target coverage is observed. The dose volume histogram (DVH) for the GTV is shifted to the left by 36 cGy for the single fraction, which would extrapolate out to a 180 cGy shift if the dose distribution was the same for all the fractions (as shown in Figure 8D). This example illustrates the importance of managing bowel gas and paying attention to large changes in gas between the simulation and treatment. Since previous treatments of this patient had involved gas levels that were similar to those that occurred during the simulation, this deviation was deemed acceptable. However, consistent changes in gas or a more dramatic change could warrant requiring the patient to return later to try again or for the application of a verification plan. In addition to unexpected loss of target coverage, the accuracy of the IDL contours used to evaluate OAR avoidance during alignment is of potential concern in similar cases. Large changes in gas near the tumor could result in changes to the IDL shape, which could result in unnecessary shifts away from GTV or higher than anticipated doses to the OARs. In this patient, the difference in the shapes of the lower dose IDLs was very small, so the OAR avoidance strategy was not changed. Noticeable unanticipated differences in doses to the targets and OARs can generally be avoided by being observant enough to notice changes in gas distribution during alignment, noting patterns in gas filling over multiple fractions when larger fractional changes are observed, and being aware of the limitations of using IDLs calculated from the simulation CT in these situations. Other solutions for mitigating the effects of gas include dietary restrictions and gas medications; overriding large, unexpected areas of gas in the planning CT with water; and delaying the treatment and recalculating the dose in regard to the daily CTOR image for verification. The amount of gas and the number of fractions affected was deemed small enough that overriding the gas and dose recalculation were not used in this case.

### 3.2. Case Study 2

**Figure 9:**
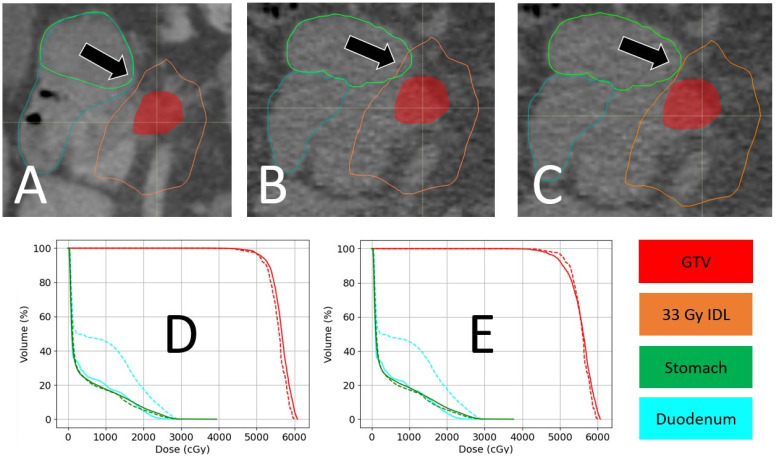
An example of pre-treatment shifting via CTOR. (**A**) Shows the treatment plan according to the simulation, with the GTV in red, stomach in green, duodenum in blue, and the 33 Gy isodose line in orange. Here, the plan was designed to stop 33 Gy from being received by the stomach and duodenum (acceptable within 0.5 cm^3^). (**B**) Shows the day-of CTOR image, which indicates that the stomach would partially receive 33 Gy near the black arrow if nothing is done. (**C**) Shows the results of manually shifting the isocenter by 3 mm laterally and 1 mm posteriorly, causing the 33 Gy isodose line to stay away from the stomach. (**D**) Compares the DVHs of the planned and day-of GTV-aligned dose distributions as a sum of all the fractions (unshifted daily dose in solid lines, planned dose in dashed lines), while (**E**) compares the planned and day-of manually shifted dose distributions as a sum of all the fractions (shifted daily dose in solid lines, planned dose in dashed lines).

The second case study examines pancreatic SBRT treatment, where anatomy changes near the target are present. This patient with adenocarcinoma of the pancreas received an SIB prescription of 50 Gy in five fractions, with a low-dose PTV prescription of 30 Gy in five fractions. The clinical goal for the duodenum (blue contour) and stomach (green contour) in regard to this treatment was V33 Gy < 0.5 cm^3^. To ensure this goal was met, a 33 Gy IDL (orange contour) was sent to the alignment software from the treatment planning system. For three out of the five fractions, a small overlap between the 33 Gy IDL and the stomach or duodenum was noted. To meet the clinical goal for these OARs, the alignment was shifted away from perfect GTV alignment, until the IDL just skimmed the edge of the OAR. Figure 9 shows the fraction with the largest difference in the anatomy (black arrow) between the simulation (Figure 9A) and treatment (Figure 9B,C) CT scan. Using the GTV to align the simulation CT to the daily CT without accounting for the OARs would have resulted in a portion of the stomach receiving 33 Gy, as seen in Figure 8B. If this strategy were to be repeated for all the fractions, the total maximum dose to the stomach would have been 39.4 Gy, with 0.15 cm^3^ receiving 33 Gy or higher (Figure 9D). However, by shifting the isocenter 3 mm laterally and 1 mm posteriorly, the 33 Gy IDL no longer overlaps with the stomach (Figure 9C). Using this strategy, the maximum dose to the stomach would be 37.7 Gy, with 0.13 cm^3^ receiving 33 Gy or higher, with a modest, but clinically acceptable, drop in GTV coverage (D95% = 97.6%).

### 3.3. Case Study 3

**Figure 10:**
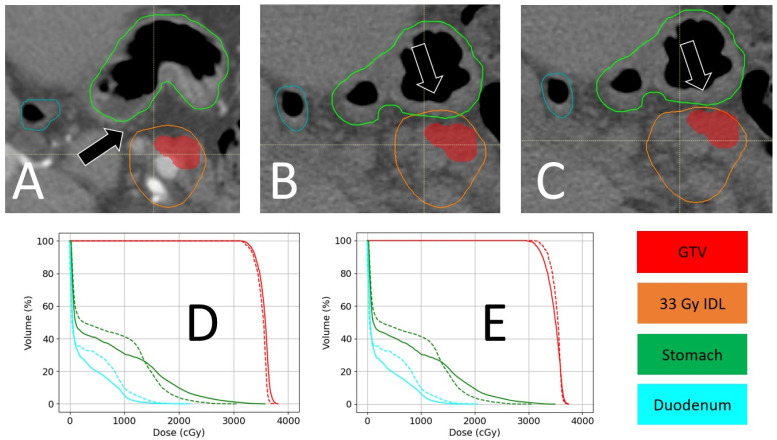
Example of pre-treatment shifting via CTOR. (**A**) Shows the treatment plan according to the simulation, with the GTV in red, stomach in green, duodenum in blue, and the 33 Gy isodose line in orange. Here, the plan was designed to stop 33 Gy from being received by the stomach and duodenum (acceptable within 0.5 cm^3^). (**B**) Shows the day-of CTOR image, which indicates that the stomach partially receives 33 Gy near the black arrow. (**C**) Shows the results of manually shifting the isocenter by 0.3 cm laterally, 0.2 cm superiorly, and 0.4 cm posteriorly, causing the 33 Gy isodose line to stay away from the stomach. (**D**) Compares the DVHs of the planned and day-of GTV-aligned dose distributions as a sum of all the fractions (unshifted daily dose in solid lines, planned dose in dashed lines), while (**E**) compares the planned and day-of manually shifted dose distributions as a sum of all the fractions (shifted daily dose in solid lines, planned dose in dashed lines).

The third case study looks at an example of a patient with a more dramatic difference in their anatomy between the simulation (Figure 10A) and treatment (Figure 10B,C) CT scans. This patient with adenocarcinoma of the pancreas received SBRT to the pancreas with a prescription of 36 Gy in five fractions, with a clinical goal being for the stomach and duodenum to be subject to a V33 Gy < 0. 5 cm^3^. For this patient, four out of five fractions required an isocenter shift to avoid the 33 Gy IDL overlapping the stomach. Aligning to the GTV and ignoring the IDL overlaps with the bowel would have resulted in total doses (Figure 10D) of 22.0 Gy and 35.7 Gy as the maximum dose and 0.0 cc and 0.41 cc > 33 Gy for the duodenum and stomach, respectively. Shifting the IDL away from the stomach, as in Figure 10C, results in total doses (Figure 10E) of 19.9 Gy and 34.9 Gy as the maximum dose and 0.0 cm^3^ and 0.25 cm^3^ > 33 Gy for the duodenum and stomach, respectively. The target coverage (D95%) in this case was reduced from 100 to 96.6%.

## 4. Discussion

This paper has described one approach to addressing the many challenges to safely delivering high doses of radiation to the abdomen and has demonstrated its validity in three patients. The high contrast and relatively low noise imaging of CT-based alignment allows for accurate GTV alignment and the evaluation of the OAR dose, based on planning CT IDLs. Slight adjustments to the alignment to avoid high doses of SBRT to OARs enable safe dose escalation, without the added complexity of daily adaptive treatments. In each of the patients examined, changes in their anatomy could lead to unexpectedly low target coverage (case 1) or unexpectedly high OAR doses (cases 2 and 3), if one were to rely solely on GTV alignment. These examples, as well as a number of more extensive studies, illustrate the consequences of treatment strategies that do not account for changing anatomy in the abdomen. With standard CBCT, it is not always possible to accurately evaluate the OAR positions due to image quality limitations, which emphasizes the importance of high-quality imaging, such as CTOR.

As is often the case in the abdomen, a balance must be struck between avoiding normal tissue toxicity and maximizing the dose to the tumor. It should be noted that the strategy discussed in this paper is conservative in terms of ensuring normal tissue sparing at the potential cost of GTV coverage. For each fraction, the assumption is that the fractional dose distribution will be the total dose distribution, even though on other days the OAR in question may be further away. It should be noted that in case studies 2 and 3, the total doses to the stomach and duodenum with GTV only alignment, while higher than with shifts away from the OARs, were still within the tolerance thresholds. However, without knowing the cumulative dose at the time of treatment, caution is warranted to ensure the tolerance thresholds are met. In the work by Niedzielski et. al., the author retrospectively calculated the cumulative dose in regard to CTOR images with simulated fiducial-only alignment, and three out of eleven patients had at least one OAR that exceeded the tolerance level [36]. While this strategy is necessarily conservative in sparing normal tissues, due to the seriousness of potential toxicity, clinical judgement should be used to ensure the proper balance is being met. Further study on the effects of alignment adjustments based on IDLs would be beneficial to better understand this tradeoff, as only two cases were examined closely in this study.

While a dosimetrically ideal solution would be to use daily online adaptive planning, the strategy demonstrated here involving the use of IDLs to shift away from OARs shows improved OAR doses with clinically acceptable overall decreases in the GTV dose compared to simply aligning with the GTV. Note that the patient case studies discussed here represent some of the larger changes seen in a patient’s anatomy that were still treatable without adaptation, and that typically smaller shifts away from the GTV are observed. Online adaptive planning is time consuming and personnel resource intensive, decreasing the overall throughput and leaving less time for physicists and physicians to work on other tasks. In regard to this workflow, physicians are only required to review images (5 to 10 min), whereas with an online adaptive workflow, they would be required to perform or approve the contouring and revised plan as well. The added time between imaging and treatment with an online adaptive workflow increases the likelihood that the patient’s internal anatomy will have shifted. Overall, the strategy presented here represents a good middle ground that can reduce the chances of overdosing OARs, without the added time required and complexity of online adaptive planning.

A limitation of using IDLs that are derived from simulation CT scans is that changes in the patient’s anatomy could affect the dose distribution. Changes are particularly likely when large differences in the amount of gas are present, as in case 1. Caution should be used when these differences are observed and strategies, such as NPO instructions and gas reduction medications, should be employed to lessen the probability of them occurring. However, note that even in the patient with a noticeable difference in the amount of gas in their system and in the target coverage area, the lower dose IDLs were very similar to the simulation and were unlikely to impact the quality of the treatment if relied on. A more extensive study would be necessary to more quantitatively study this effect.

A key aspect of safely delivering high doses of SBRT to tumors that are close to GI luminal structures is sufficient image quality to be able to visualize both the GTV and the surrounding OARs, particularly at their borders. As can be seen from the CTOR images in this paper, this treatment modality has sufficient image quality to visualize both the targets (or nearby surrogates) and the OARs. Traditionally, artifacts and relatively poor contrast have made such visualization difficult in CBCT images, especially in larger patients or patients with implanted metal or excessive gas. However, improvements in CBCT imaging are closing the gap between diagnostic-quality CT and CBCT, potentially allowing for similar strategies to be applied to CBCT-based SBRT [65,66,67,68,69]. While the specialized technology described in this paper is of limited availability, the strategies acquired from our institution’s extensive experience with CTOR and the methods described in this paper can help to lower the human expertise gap in implementing these commercially available products.

The motion management in terms of inhale BH gating with RPM-based motion tracking that is described in this paper should be understood as one of several valid options for dealing with respiratory motion. Other strategies include exhale BH, compression belts, gating, or 4DCT-based ITV. However, the user should use caution when opting for free-breathing strategies that use CTOR (or fast CBCT) to avoid aligning to the tumor when it is in the extreme inhale or exhale phase.

A limitation of this study is that the low number of patients examined does not allow for definitive conclusions about the dose distributions resulting from shifting away from OARs and how these compare to CBCT-based alignment. A full quantitative analysis of this strategy is an area in need of future work. However, evidence exists for the exceedance of OAR tolerances in the abdomen when the alignment focus is only on the GTV [36]. In both our work and the work by Niedzielski et al., the assumption in comparing to CBCT-based alignment is that CBCT alignment is solely based on fiducial markers. This oversimplification ignores the fact that OARs can been seen on CBCT images in some patients and, thus, be avoided. Another area of future work is to review patient outcomes from this treatment approach and compare them to standard-of-care methods to better understand the clinical benefits of this technique.

## 5. Conclusions

High-quality daily imaging for abdominal SBRT allows the adequate visualization of both the tumor and organs at risk (OARs). A clear OAR delineation allows for the use of isodose line contours to shift high-dose regions away from OARs. Accurate target localization and OAR avoidance enables safe dose escalation, which can help to more effectively treat challenging tumors.

## Figures and Tables

**Table 1:** Relative comparison of methods used to avoid exceeding GI organ-at-risk (OAR) tolerance during the delivery of SBRT to the abdomen.

Approach	Risk to OARs	Treatment Efficacy	Complexity of Treatment	Physician Time Requirement	Requires High Quality Imaging
Lower prescription dose	low	low	low	low	no
Use IDLs to avoid OARs	med	med	low	med	yes
Offline adaptation	med	med	med	high	yes
Online adaptation	low	high	high	high	yes

## Data Availability

The raw data supporting the conclusions of this article will be made available by the authors on request.

## References

[1] Bouchart C., Navez J., Closset J., Hendlisz A., Van Gestel D., Moretti L., Van Laethem J.-L. (2020). Novel strategies using modern radiotherapy to improve pancreatic cancer outcomes: Toward a new standard?. Ther. Adv. Med. Oncol..

[2] Arcelli A., Guido A., Buwenge M., Simoni N., Mazzarotto R., Macchia G., Deodato F., Cilla S., Bonomo P., Scotti V. (2020). Higher Biologically Effective Dose Predicts Survival in SBRT of Pancreatic Cancer: A Multicentric Analysis (PAULA-1). Anticancer Res..

[3] Cellini F., Arcelli A., Simoni N., Caravatta L., Buwenge M., Calabrese A., Brunetti O., Genovesi D., Mazzarotto R., Deodato F. (2020). Basics and Frontiers on Pancreatic Cancer for Radiation Oncology: Target Delineation, SBRT, SIB Technique, MRgRT, Particle Therapy, Immunotherapy and Clinical Guidelines. Cancers.

[4] Chang D.T., Schellenberg D., Shen J., Kim J., Goodman K.A., Fisher G.A., Ford J.M., Desser T., Quon A., Koong A.C. (2009). Stereotactic radiotherapy for unresectable adenocarcinoma of the pancreas. Cancer.

[5] Chen-Zhao X., Hernando O., López M., Sánchez E., Montero A., García-Aranda M., Ciérvide R., Valero J., Alonso R., Cárdenas-Rebollo J.M. (2020). A prospective observational study of the clinical and pathological impact of stereotactic body radiotherapy (SBRT) as a neoadjuvant strategy of chemoradiation in pancreatic cancer. Clin. Transl. Oncol..

[6] Dalwadi S.M., Herman J.M., Das P., Holliday E.B. (2020). Novel Radiotherapy Technologies in the Treatment of Gastrointestinal Malignancies. Hematol. Oncol. Clin. N. Am..

[7] Mahadevan A., Jain S., Goldstein M., Miksad R., Pleskow D., Sawhney M., Brennan D., Callery M., Vollmer C. (2010). Stereotactic Body Radiotherapy and Gemcitabine for Locally Advanced Pancreatic Cancer. Int. J. Radiat. Oncol..

[8] Mellon E.A., Hoffe S.E., Springett G.M., Frakes J.M., Strom T.J., Hodul P.J., Malafa M.P., Chuong M.D., Shridhar R. (2015). Long-term outcomes of induction chemotherapy and neoadjuvant stereotactic body radiotherapy for borderline resectable and locally advanced pancreatic adenocarcinoma. Acta Oncol..

[9] Petrelli F., Comito T., Ghidini A., Torri V., Scorsetti M., Barni S. (2017). Stereotactic Body Radiation Therapy for Locally Advanced Pancreatic Cancer: A Systematic Review and Pooled Analysis of 19 Trials. Int. J. Radiat. Oncol..

[10] Reyngold M., Parikh P., Crane C.H. (2019). Ablative radiation therapy for locally advanced pancreatic cancer: Techniques and results. Radiat. Oncol..

[11] Zhong J., Patel K., Switchenko J., Cassidy R.J., Hall W.A., Gillespie T., Patel P.R., Kooby D., Landry J. (2017). Outcomes for patients with locally advanced pancreatic adenocarcinoma treated with stereotactic body radiation therapy versus conventionally fractionated radiation. Cancer.

[12] Palta M., Godfrey D., Goodman K.A., Hoffe S., Dawson L.A., Dessert D., Hall W.A., Herman J.M., Khorana A.A., Merchant N. (2019). Radiation Therapy for Pancreatic Cancer: Executive Summary of an ASTRO Clinical Practice Guideline. Pract. Radiat. Oncol..

[13] Herman J.M., Chang D.T., Goodman K.A., Dholakia A.S., Raman S.P., Hacker-Prietz A., Iacobuzio-Donahue C.A., Griffith M.E., Pawlik T.M., Pai J.S. (2015). Phase 2 multi-institutional trial evaluating gemcitabine and stereotactic body radiotherapy for patients with locally advanced unresectable pancreatic adenocarcinoma. Cancer.

[14] Poon R.T.-P., Fan S.-T., Lo C.-M., Ng I.O.-L., Liu C.-L., Lam C.-M., Wong J. (2001). Improving Survival Results After Resection of Hepatocellular Carcinoma: A Prospective Study of 377 Patients Over 10 Years. Ann. Surg..

[15] Mornex F., Girard N., Beziat C., Kubas A., Khodri M., Trepo C., Merle P. (2006). Feasibility and efficacy of high-dose three-dimensional-conformal radiotherapy in cirrhotic patients with small-size hepatocellular carcinoma non-eligible for curative therapies—Mature results of the French Phase II RTF-1 trial. Int. J. Radiat. Oncol..

[16] Ben-Josef E., Normolle D., Ensminger W.D., Walker S., Tatro D., Ten Haken R.K., Knol J., Dawson L.A., Pan C., Lawrence T.S. (2005). Phase II Trial of High-Dose Conformal Radiation Therapy With Concurrent Hepatic Artery Floxuridine for Unresectable Intrahepatic Malignancies. J. Clin. Oncol..

[17] Skinner H.D., Sharp H.J., Kaseb A.O., Javle M.M., Vauthey J.N., Abdalla E.K., Delclos M.E., Das P., Crane C.H., Krishnan S. (2011). Radiation treatment outcomes for unresectable hepatocellular carcinoma. Acta Oncol..

[18] Tao R., Krishnan S., Bhosale P.R., Javle M.M., Aloia T.A., Shroff R.T., Kaseb A.O., Bishop A.J., Swanick C.W., Koay E.J. (2016). Ablative Radiotherapy Doses Lead to a Substantial Prolongation of Survival in Patients With Inoperable Intrahepatic Cholangiocarcinoma: A Retrospective Dose Response Analysis. J. Clin. Oncol..

[19] McPartlin A., Swaminath A., Wang R., Pintilie M., Brierley J., Kim J., Ringash J., Wong R., Dinniwell R., Craig T. (2017). Long-Term Outcomes of Phase 1 and 2 Studies of SBRT for Hepatic Colorectal Metastases. Int. J. Radiat. Oncol..

[20] Chang B.K., Timmerman R.D. (2007). Stereotactic Body Radiation Therapy: A Comprehensive Review. Am. J. Clin. Oncol..

[21] Grimbergen G., Eijkelenkamp H., Heerkens H.D., Raaymakers B.W., Intven M.P.W., Meijer G.J. (2022). Intrafraction pancreatic tumor motion patterns during ungated magnetic resonance guided radiotherapy with an abdominal corset. Phys. Imaging Radiat. Oncol..

[22] Ge J., Santanam L., Noel C., Parikh P.J. (2013). Planning 4-Dimensional Computed Tomography (4DCT) Cannot Adequately Represent Daily Intrafractional Motion of Abdominal Tumors. Int. J. Radiat. Oncol..

[23] Marchant T.E., Amer A.M., Moore C.J. (2008). Measurement of inter and intra fraction organ motion in radiotherapy using cone beam CT projection images. Phys. Med. Biol..

[24] Minn A.Y., Schellenberg D., Maxim P., Suh Y., McKenna S., Cox B., Dieterich S., Xing L., Graves E., Goodman K.A. (2009). Pancreatic Tumor Motion on a Single Planning 4D-CT Does Not Correlate With Intrafraction Tumor Motion During Treatment. Am. J. Clin. Oncol..

[25] Rusu D.N., Cunningham J.M., Arch J.V., Chetty I.J., Parikh P.J., Dolan J.L. (2023). Impact of intrafraction motion in pancreatic cancer treatments with MR-guided adaptive radiation therapy. Front. Oncol..

[26] Hooshangnejad H., Miles D., Hill C., Narang A., Ding K., Han-Oh S. (2023). Inter-Breath-Hold Geometric and Dosimetric Variations in Organs at Risk during Pancreatic Stereotactic Body Radiotherapy: Implications for Adaptive Radiation Therapy. Cancers.

[27] Keall P.J. (2006). The management of respiratory motion in radiation oncology report of AAPM Task Group 76a. Med. Phys..

[28] Young T., Lee M., Johnston M., Nguyen T., Ko R., Arumugam S. (2023). Assessment of interfraction dose variation in pancreas SBRT using daily simulation MR images. Phys. Eng. Sci. Med..

[29] Beddar A.S., Briere T.M., Balter P., Pan T., Tolani N., Ng C., Szklaruk J., Krishnan S. (2008). 4D-CT imaging with synchronized intravenous contrast injection to improve delineation of liver tumors for treatment planning. Radiother. Oncol..

[30] Eccles C.L., Tse R.V., Hawkins M.A., Lee M.T., Moseley D.J., Dawson L.A. (2016). Intravenous contrast-enhanced cone beam computed tomography (IVCBCT) of intrahepatic tumors and vessels. Adv. Radiat. Oncol..

[31] Daamen L.A., Parikh P.J., Hall W.A. (2024). The Use of MR-Guided Radiation Therapy for Pancreatic Cancer. Semin. Radiat. Oncol..

[32] Gough J., Hall W., Good J., Nash A., Aitken K. (2022). Technical Radiotherapy Advances—The Role of Magnetic Resonance Imaging-Guided Radiation in the Delivery of Hypofractionation. Clin. Oncol..

[33] Prime S., Schiff J.P., Hosni A., Stanescu T., Dawson L.A., Henke L.E. (2024). The Use of MR-Guided Radiation Therapy for Liver Cancer. Semin. Radiat. Oncol..

[34] Breazeale A., Rahmani R., Gallagher K., Nabavizadeh N. (2024). Liver stereotactic body radiation therapy without fiducial or retained ethiodized oil guidance warrants greater than 5 mm planning target volumes. J. Med. Radiat. Sci..

[35] Moskalenko M., Jones B.L., Mueller A., Lewis S., Shiao J.C., Zakem S.J., Robin T.P., Goodman K.A. (2023). Fiducial Markers Allow Accurate and Reproducible Delivery of Liver Stereotactic Body Radiation Therapy. Curr. Oncol..

[36] Niedzielski J.S., Liu Y., Ng S.S.W., Martin R.M., Perles L.A., Beddar S., Rebueno N., Koay E.J., Taniguchi C., Holliday E.B. (2021). Dosimetric Uncertainties Resulting From Interfractional Anatomic Variations for Patients Receiving Pancreas Stereotactic Body Radiation Therapy and Cone Beam Computed Tomography Image Guidance. Int. J. Radiat. Oncol..

[37] Hammers J., Lindsay D., Narayanasamy G., Sud S., Tan X., Dooley J., Marks L.B., Chen R.C., Das S.K., Mavroidis P. (2023). Evaluation of the clinical impact of the differences between planned and delivered dose in prostate cancer radiotherapy based on CT-on-rails IGRT and patient-reported outcome scores. J. Appl. Clin. Med. Phys..

[38] Li X., Quan E.M., Li Y., Pan X., Zhou Y., Wang X., Du W., Kudchadker R.J., Johnson J.L., Kuban D.A. (2013). A Fully Automated Method for CT-on-Rails-Guided Online Adaptive Planning for Prostate Cancer Intensity Modulated Radiation Therapy. Int. J. Radiat. Oncol..

[39] Ma C.-M.C., Paskalev K. (2006). In-room CT techniques for image-guided radiation therapy. Med. Dosim..

[40] Owen R., Kron T., Foroudi F., Milner A., Cox J., Duchesne G., Cleeve L., Zhu L., Cramb J., Sparks L. (2009). Comparison of CT on Rails With Electronic Portal Imaging for Positioning of Prostate Cancer Patients With Implanted Fiducial Markers. Int. J. Radiat. Oncol..

[41] Yang Z., Chang Y., Brock K.K., Cazoulat G., Koay E.J., Koong A.C., Herman J.M., Park P.C., Poenisch F., Li Q. (2019). Effect of setup and inter-fraction anatomical changes on the accumulated dose in CT-guided breath-hold intensity modulated proton therapy of liver malignancies. Radiother. Oncol..

[42] Bohoudi O., Bruynzeel A.M.E., Meijerink M.R., Senan S., Slotman B.J., Palacios M.A., Lagerwaard F.J. (2019). Identification of patients with locally advanced pancreatic cancer benefitting from plan adaptation in MR-guided radiation therapy. Radiother. Oncol..

[43] Chuong M.D., Bryant J., Mittauer K.E., Hall M., Kotecha R., Alvarez D., Romaguera T., Rubens M., Adamson S., Godley A. (2021). Ablative 5-Fraction Stereotactic Magnetic Resonance–Guided Radiation Therapy With On-Table Adaptive Replanning and Elective Nodal Irradiation for Inoperable Pancreas Cancer. Pract. Radiat. Oncol..

[44] Doty D.G., Chuong M.D., Gomez A.G., Bryant J., Contreras J., Romaguera T., Alvarez D., Kotecha R., Mehta M.P., Gutierrez A.N. (2021). Stereotactic MR-guided online adaptive radiotherapy reirradiation (SMART reRT) for locally recurrent pancreatic adenocarcinoma: A case report. Med. Dosim..

[45] Ermongkonchai T., Khor R., Muralidharan V., Tebbutt N., Lim K., Kutaiba N., Ng S.P. (2022). Stereotactic radiotherapy and the potential role of magnetic resonance-guided adaptive techniques for pancreatic cancer. World J. Gastroenterol..

[46] Hawranko R., Sohn J.J., Neiderer K., Bump E., Harris T., Fields E.C., Weiss E., Song W.Y. (2022). Investigation of Isotoxic Dose Escalation and Plan Quality with TDABC Analysis on a 0.35 T MR-Linac (MRL) System in Ablative 5-Fraction Stereotactic Magnetic Resonance-Guided Radiation Therapy (MRgRT) for Primary Pancreatic Cancer. J. Clin. Med..

[47] Herr D.J., Wang C., Mendiratta-Lala M., Matuszak M., Mayo C.S., Cao Y., Parikh N.D., Ten Haken R., Owen D., Evans J.R. (2023). A Phase II Study of Optimized Individualized Adaptive Radiotherapy for Hepatocellular Carcinoma. Clin. Cancer Res..

[48] Hill C.S., Han-Oh S., Cheng Z., Wang K.K.-H., Meyer J.J., Herman J.M., Narang A.K. (2021). Fiducial-based image-guided SBRT for pancreatic adenocarcinoma: Does inter-and intra-fraction treatment variation warrant adaptive therapy?. Radiat. Oncol..

[49] Lee D., Renz P., Oh S., Hwang M.-S., Pavord D., Yun K.L., Collura C., McCauley M., Colonias A., Trombetta M. (2023). Online Adaptive MRI-Guided Stereotactic Body Radiotherapy for Pancreatic and Other Intra-Abdominal Cancers. Cancers.

[50] Magallon-Baro A., Granton P.V., Milder M.T.W., Loi M., Zolnay A.G., Nuyttens J.J., Hoogeman M.S. (2019). A model-based patient selection tool to identify who may be at risk of exceeding dose tolerances during pancreatic SBRT. Radiother. Oncol..

[51] Mittauer K.E., Yarlagadda S., Bryant J.M., Bassiri N., Romaguera T., Gomez A.G., Herrera R., Kotecha R., Mehta M.P., Gutierrez A.N. (2023). Online adaptive radiotherapy: Assessment of planning technique and its impact on longitudinal plan quality robustness in pancreatic cancer. Radiother. Oncol..

[52] Rhee D.J., Beddar S., Jaoude J.A., Sawakuchi G., Martin R., Perles L., Yu C., He Y., Court L.E., Ludmir E.B. (2023). Dose Escalation for Pancreas SBRT: Potential and Limitations of using Daily Online Adaptive Radiation Therapy and an Iterative Isotoxicity Automated Planning Approach. Adv. Radiat. Oncol..

[53] Prasad Venkatesulu B., Ness E., Ross D., Saripalli A.L., Abood G., Badami A., Cotler S., Dhanarajan A., Knab L.M., Lee B. (2023). MRI-guided Real-time Online Gated Stereotactic Body Radiation Therapy for Liver Tumors. Am. J. Clin. Oncol..

[54] Weykamp F., Katsigiannopulos E., Piskorski L., Regnery S., Hoegen P., Ristau J., Renkamp C.K., Liermann J., Forster T., Lang K. (2022). Dosimetric Benefit of Adaptive Magnetic Resonance-Guided Stereotactic Body Radiotherapy of Liver Metastases. Cancers.

[55] Wu T.C., Yoon S.M., Cao M., Raldow A.C., Xiang M. (2023). Identifying predictors of on-table adaptation for pancreas stereotactic body radiotherapy (SBRT). Clin. Transl. Radiat. Oncol..

[56] Chuong M.D., Clark M.A., Henke L.E., Kishan A.U., Portelance L., Parikh P.J., Bassetti M.F., Nagar H., Rosenberg S.A., Mehta M.P. (2023). Patterns of utilization and clinical adoption of 0.35 Tesla MR-guided radiation therapy in the United States—Understanding the transition to adaptive, ultra-hypofractionated treatments. Clin. Transl. Radiat. Oncol..

[57] Garcia Schüler H.I., Pavic M., Mayinger M., Weitkamp N., Chamberlain M., Reiner C., Linsenmeier C., Balermpas P., Krayenbühl J., Guckenberger M. (2021). Operating procedures, risk management and challenges during implementation of adaptive and non-adaptive MR-guided radiotherapy: 1-year single-center experience. Radiat. Oncol..

[58] Sanders M.K., Moser A.J., Khalid A., Fasanella K.E., Zeh H.J., Burton S., McGrath K. (2010). EUS-guided fiducial placement for stereotactic body radiotherapy in locally advanced and recurrent pancreatic cancer. Gastrointest. Endosc..

[59] Krishnan S., Briere T.M., Dong L., Murthy R., Ng C., Balter P., Mohan R., Gillin M.T., Beddar A.S. (2007). Daily targeting of liver tumors: Screening patients with a mock treatment and using a combination of internal and external fiducials for image-guided respiratory-gated radiotherapya. Med. Phys..

[60] Slagowski J.M., Colbert L.E., Cazacu I.M., Singh B.S., Martin R., Koay E.J., Taniguchi C.M., Koong A.C., Bhutani M.S., Herman J.M. (2020). Evaluation of the Visibility and Artifacts of 11 Common Fiducial Markers for Image Guided Stereotactic Body Radiation Therapy in the Abdomen. Pract. Radiat. Oncol..

[61] Koay E.J., Hanania A.N., Hall W.A., Taniguchi C.M., Rebueno N., Myrehaug S., Aitken K.L., Dawson L.A., Crane C.H., Herman J.M. (2020). Dose-Escalated Radiation Therapy for Pancreatic Cancer: A Simultaneous Integrated Boost Approach. Pract. Radiat. Oncol..

[62] Simoni N., Micera R., Paiella S., Guariglia S., Zivelonghi E., Malleo G., Rossi G., Addari L., Giuliani T., Pollini T. (2021). Hypofractionated Stereotactic Body Radiation Therapy with Simultaneous Integrated Boost and Simultaneous Integrated Protection in Pancreatic Ductal Adenocarcinoma. Clin. Oncol..

[63] Zhang L., Dong L., Court L., Wang H., Gillin M., Mohan R. (2005). TU-EE-A4-05: Validation of CT-Assisted Targeting (CAT) Software for Soft Tissue and Bony Target Localization. Med. Phys..

[64] Benedict S.H., Yenice K.M., Followill D., Galvin J.M., Hinson W., Kavanagh B., Keall P., Lovelock M., Meeks S., Papiez L. (2010). Stereotactic body radiation therapy: The report of AAPM Task Group 101. Med. Phys..

[65] Robar J.L., Cherpak A., MacDonald R.L., Yashayaeva A., McAloney D., McMaster N., Zhan K., Cwajna S., Patil N., Dahn H. (2024). Novel Technology Allowing Cone Beam Computed Tomography in 6 Seconds: A Patient Study of Comparative Image Quality. Pract. Radiat. Oncol..

[66] Zhang Q., Hu Y., Liu F., Goodman K., Rosenzweig K.E., Mageras G.S. (2010). Correction of motion artifacts in cone-beam CT using a patient-specific respiratory motion model. Med. Phys..

[67] Dai X., Lei Y., Wynne J., Janopaul-Naylor J., Wang T., Roper J., Curran W.J., Liu T., Patel P., Yang X. (2021). Synthetic CT-aided multiorgan segmentation for CBCT-guided adaptive pancreatic radiotherapy. Med. Phys..

[68] Gao L., Xie K., Sun J., Lin T., Sui J., Yang G., Ni X. (2023). Streaking artifact reduction for CBCT-based synthetic CT generation in adaptive radiotherapy. Med. Phys..

[69] Hrinivich W.T., Chernavsky N.E., Morcos M., Li T., Wu P., Wong J., Siewerdsen J.H. (2022). Effect of subject motion and gantry rotation speed on image quality and dose delivery in CT-guided radiotherapy. Med. Phys..

